# How Insects Balance Reproductive Output and Immune Investment

**DOI:** 10.3390/insects16030311

**Published:** 2025-03-17

**Authors:** Jimena Leyria, Leonardo L. Fruttero, Pedro A. Paglione, Lilián E. Canavoso

**Affiliations:** 1 Departamento de Bioquímica Clínica, Facultad de Ciencias Químicas, Universidad Nacional de Córdoba, Córdoba 5000, CP, Argentina; jimena.leyria@unc.edu.ar (J.L.); lfruttero@unc.edu.ar (L.L.F.); pedro.paglione@unc.edu.ar (P.A.P.); 2 Centro de Investigaciones en Bioquímica Clínica e Inmunología (CIBICI), Consejo Nacional de Investigaciones Científicas y Técnicas (CONICET), Córdoba 5000, CP, Argentina

**Keywords:** insect reproduction, insect immune response, energy-demanding process, energy allocation, trade-off, neuroendocrine/endocrine regulation, juvenile hormone, ecdysteroids, vitellogenin, lipophorin

## Abstract

Insects face the constant challenge of balancing energy allocation between reproduction and immune responses, both of which are highly energy-demanding processes. Immune challenges frequently result in decreased fecundity, reduced egg viability, and delayed ovarian development. Conversely, heightened reproductive activity often suppresses immune functions. This trade-off has profound ecological and evolutionary consequences, shaping insects’ survival, adaptation, and population dynamics. The intricate interplay between reproduction and immunity in insects is regulated by the neuroendocrine and endocrine systems, which orchestrate resource distribution alongside other biological processes. Key hormones, such as juvenile hormone and ecdysteroids, serve as central regulators, influencing both immune responses and reproductive activities. Additionally, macromolecules like vitellogenin and lipophorin, primarily known for their functions as yolk protein precursors and lipid carriers, play crucial roles in pathogen recognition and transgenerational immune priming. Advancements in molecular and omics tools have unveiled the complexity of these regulatory mechanisms, providing new insights into how insects dynamically allocate resources to optimize their fitness. This delicate balance underscores critical evolutionary strategies and the integration of physiological systems across species. This review synthesizes insights from life history theory, oogenesis, and immunity, offering new perspectives on the trade-offs between reproductive output and immune investment.

## Simple Summary

Female insects face a tough decision: how to allocate their limited energy between reproducing and fighting off infections. Both processes are energy-demanding, so prioritizing one often comes at the expense of the other. This delicate balance affects their survival, ability to adapt, and population dynamics. Hormones and other biological signals play a key role in determining how energy is utilized. This review examines how a female insect’s immune system influences its reproductive capacity and discusses the mechanisms behind this trade-off. It also highlights gaps in current knowledge and suggests directions for future studies. Recent advances in research tools have revealed the intricate nature of these systems, showing how insects balance immunity and reproduction to survive. Understanding these strategies contributes to addressing important challenges, such as controlling pest insects or protecting beneficial ones. This knowledge also offers fascinating insights into evolution and the fine balance that sustains life.

## 1. Introduction

All processes in an individual’s life are governed by the allocation of energy and resources. Life history theory provides a framework to understand this interplay [1], particularly under challenging scenarios such as food scarcity, egg production, or the need to combat infections. Insects face particularly demanding trade-offs, as reproduction and immunity are both metabolically costly processes. Insect reproduction involves complex and energy-demanding events, including mate selection, courtship behaviors, copulation, egg production (oogenesis), fertilization, and oviposition [2]. Similarly, immune responses require significant metabolic investment to support the activation and functioning of signaling pathways, including Toll, immune deficiency (Imd), Janus kinase–signal transducer and activator of transcription (Jak-STAT), and c-Jun N-terminal kinase (Jnk) [3]. These pathways activate key immune effectors such as antimicrobial peptides (AMPs), phenoloxidase (PO), and reactive oxygen species (ROS), all of which are essential for combating infections. In these scenarios, female insects face a crucial trade-off: should they allocate resources to egg production, or instead prioritize mounting an immune response? This compromise is often reflected in reduced fecundity, decreased egg viability, and delayed ovarian development. Experimental studies have shown that immune challenges exacerbate these effects with pathogenic infections, which require stronger immune responses and thereby intensify the trade-off. Conversely, increased reproductive activity can suppress immune functions, further highlighting the intricate interplay between these processes [4]. This relationship has significant ecological and evolutionary implications, influencing insects’ survival, adaptation, and population dynamics. Understanding the mechanisms underlying this trade-off provides valuable insights into pest control and conservation strategies, emphasizing the importance of balancing reproductive success and immune competence across diverse environmental contexts. The interplay between reproduction and immune investment in insects is regulated by their neuroendocrine and endocrine systems, which mediate physiological processes such as the resource allocation between survival and species propagation. Juvenile hormone (JH) and ecdysteroids, particularly 20-hydroxyecdysone (20E), play pivotal roles as regulators, influencing both immune responses and reproductive processes [5,6]. Additionally, mating and nutritional signals further modulate immune and reproductive functions [7,8]. Mating often suppresses immune responses in females, reallocating resources to vitellogenesis while increasing vulnerability to infections. Insulin-like peptides (ILPs) further contribute by regulating hemocyte proliferation and egg development, thereby balancing immune and reproductive priorities [5,6,9,10]. Vitellogenin and lipophorin are essential yolk protein precursors for egg development in insects [11,12]. Beyond this role, vitellogenin, a phospholipoglycoprotein, serves in immunity by binding and neutralizing microbial components, while lipophorin primarily facilitates lipid transport but also functions as a pattern recognition molecule, aiding in the insect’s immune response [13].

In a recent review, Cardoso-Jaime et al. [14] analyzed the importance of essential nutritional elements such as metal ions, particularly in the immunity and reproduction of both hematophagous and non-hematophagous insects. The authors highlighted how insects optimize those physiological processes through proteins, which serve as a double-edged sword in balancing functions that protect the insect while ensuring reproductive success.

In this review, we summarize key concepts related to life history theory, oogenesis, and immune response. Additionally, we provide updated insights into how immunity influences reproductive output and the underlying mechanisms driving this relationship, with a focus on neuroendocrine and endocrine regulation of reproduction in female insects rather than males. Moreover, our central focus is on solitary insects, as their life history strategies and trade-offs between immunity and reproduction differ significantly from those of social insects. We incorporate some information on social insects for comparative purposes, as their distinctive systems offer valuable insights into the evolutionary pressures shaping immunity and reproduction across insects. In social insects, such as ants, bees, and termites, reproductive roles are often divided among castes, with queens or reproductive individuals specializing in reproduction, while workers prioritize colony maintenance and defense. This division of labor creates unique dynamics in resource allocation, immune function, and reproductive investment that are not directly comparable to those in solitary or non-social insects. For example, in social insects, workers often exhibit heightened immune responses to protect the colony, while reproductive individuals may rely on social immunity (e.g., grooming, antimicrobial secretions) rather than individual immune defenses [15]. In contrast, solitary insects must balance immunity and reproduction within a single organism, leading to direct trade-offs between these functions.

## 2. Energy Allocation and Life History Theory

Animals obtain energy by ingesting and metabolizing food, which is subsequently allocated to essential processes such as survival, growth, and reproduction. During periods of food scarcity or adverse environmental conditions, physiological processes compete for limited energy, leading to trade-offs [16,17]. Life history theory, grounded in evolutionary biology, offers a framework to analyze how organisms maximize their reproductive success. It elucidates how energy is distributed between survival and reproduction to optimize fitness, ultimately ensuring the transmission of genetic material to subsequent generations [18,19]. For example, in the higher dipteran *Drosophila melanogaster*, increased reproductive effort often shortens lifespan, as females that allocate more resources to egg production experience accelerated aging and higher mortality rates [20].

On the other hand, excess resources can be stored for future use. According to the “allocation rule”, resources invested in one function are no longer available for another [21]. In a hypothetical world with unlimited resources, the optimal life history strategy would involve reproducing at a high rate from birth and continuing indefinitely [22]. However, physiological and ecological constraints make such a strategy impossible. Instead, organisms allocate their limited resources within these constraints to maximize their reproductive value. For instance, in the hematophagous females of the kissing bug *Rhodnius prolixus*, the digestion rate of intestinal contents is positively correlated with egg production. Thus, virgin females, in contrast to mated ones, release blood stored in the anterior midgut into the posterior midgut at a slower pace, thereby conserving nutrients. This adaptation can be seen as a biological strategy that enhances the survival of virgin females, potentially increasing their chances of reproductive success over time [23]. Nonetheless, the reduced lifespan of mated females may result from the high energy expenditure associated with egg production and the negative impact of sexual harassment by males, which further diminishes female longevity [24]. In this context, while the concept that “reproduction is costly” is widely accepted for solitary insects, an opposing view exists in social insects, where reproduction may be beneficial for longevity [24].

Efforts are currently focused on identifying “biological actors” that influence decision-making processes related to energy allocation. For example, a study using female locusts as a model demonstrated that silencing *fatty acid synthase 2* (FAS2), a key gene involved in lipid synthesis and insect energy metabolism, shifted energy allocation toward immune system activation, while significantly reducing reproductive investment [25]. Moreover, in mosquitoes, it has been proposed that ecdysone signaling mediates the trade-off between immunity and reproduction by suppressing amyloid formation [26].

The terminal investment hypothesis suggests that, when future reproduction is at risk, organisms prioritize current reproduction. In *D. melanogaster*, dietary protein plays a key role in this response. Females exposed to *Pseudomonas aeruginosa* infection increased their reproductive investment differently depending on their diet: those on a high-protein diet laid more eggs, whereas those on a standard diet improved their egg viability. This highlights the influence of protein on how flies allocate resources during immune challenges [27]. Moreover, the terminal investment threshold can vary with age. For example, in the cricket *Gryllodes sigillatus*, older males exhibit terminal investment by increasing their calling effort—a pre-copulatory reproductive trait—when exposed to moderate and high doses of an infection cue, whereas younger males do not show this response at any dose. This finding highlights that older males face higher mortality risks and, therefore, prioritize current reproduction [28]. Similarly, in the flour beetle *Tenebrio molitor*, males invest more in spermatophores, producing larger, nutrient-rich ejaculates compared to healthy individuals, as part of a terminal investment strategy. This behavior aligns with the concept that individuals facing a heightened risk of mortality prioritize immediate reproductive opportunities over future survival, enhancing their reproductive investment when their prospects for future reproduction are diminished [29].

Additionally, other mechanisms related to energy allocation may interact with the trade-off between immunity and reproduction. For instance, reduced locomotor activity in mated *R. prolixus* females represents a resource-saving strategy, as a significant portion of the ingested blood meal is already allocated to egg production. This observation supports the hypothesis that the level of locomotor activity reflects the status of nutrient reserves [23].

## 3. Reproduction

The success of insects as a group can largely be attributed to their high reproductive rates and diverse adaptations for survival and mating [24,30]. In most insects, reproduction is sexual and oviparous. After mating, the female’s eggs are fertilized by sperm from the male, and these fertilized eggs are typically deposited near a suitable food source [31].

For an egg to fully develop from a germ cell during oogenesis, vitellogenesis must occur successfully. This process is regulated by neuroendocrine/endocrine and nutritional signals, which stimulate the fat body to produce large quantities of yolk protein precursors, primarily vitellogenin. Given its complexity, vitellogenesis is an energy-demanding process, as the egg must be provisioned with all of the nutrients required for the embryo’s development independent of the maternal body [6,32].

Vitellogenin primarily serves two roles in reproduction: as a building block for the proteins that constitute the egg, and as a lipid transporter. After undergoing several post-translational modifications in the fat body, vitellogenin is secreted into the hemolymph, where it circulates until reaching the oocyte. At the plasma membrane of the oocyte, it is taken up through receptor-mediated endocytosis. Once internalized, vitellogenin, now termed “vitellin”, is stored in yolk granules, specialized lysosomes that remain inactive until the onset of embryogenesis [33].

Lipophorin is characterized by a higher lipid content and lower density in comparison to vitellogenin. Its main role is to transport and deliver lipids from their sites of synthesis or absorption to the tissues where they are utilized. During reproduction, lipophorin supplies lipids to developing oocytes through both endocytic and non-endocytic pathways [34,35].

Most processes involved in vitellogenesis and oogenesis are regulated by hormones and neuropeptides, with ILPs, JH, and ecdysteroids being the most significant. Many of these hormones, neuropeptides, and other biological neuromodulators involved in insect metabolism are synthesized in the central nervous system and associated glands, such as the *corpora allata* and *corpora cardiaca.* Moreover, the fat body and ovaries serve as endocrine tissues, as they can synthesize several hormones and neuropeptides [6,36].

## 4. Immunity

While reproduction is fundamental to the development and evolution of insects, immunity is critical for their survival and success as a group. Throughout their evolutionary history, insects have developed an effective innate immune system to combat and overcome pathogen invasions. When pathogens breach the insect’s resilient body surface, the first line of defense, and infiltrate the hemolymph, they encounter the second line of defense: an immune response comprising both cellular and humoral components [37,38,39]. However, the distinction between cellular and humoral immune reactions is not always clear-cut, as many humoral factors influence hemocyte functions, and hemocytes are a significant source of various humoral molecules [40].

Pathogen-associated molecular patterns (PAMPs) such as diaminopimelic acid-type peptidoglycan (DAP-PGN), from foreign invaders, are recognized by pattern recognition receptors (PRRs) present in immune cells and tissues, including hemocytes and the fat body. Additionally, some recognition proteins, such as peptidoglycan recognition protein short (PGRP-S) and Gram-negative binding protein 3 (GNBP-3), are host molecules that bind to PAMPs to initiate an immune response. The binding of PAMPs to PRRs triggers the activation of cellular responses, including phagocytosis, nodulation, encapsulation, and melanization, and initiates signaling pathways that induce the expression of immune effector genes to counter infections. Melanization can be triggered locally in response to cuticle injury, or systemically following microbial invasion of the hemocoel [41]. Immune effectors in insects include AMPs, lectins, PO (a key enzyme in melanin biosynthesis, involved in melanization processes), ROS, nitric oxide (NO), antiviral factors, and cytokines, among others [40,42].

Recognition of pathogen-associated molecular patterns activates several signaling pathways, including Toll, Imd, Jak-STAT, and Jnk. The Toll pathway is primarily activated in response to fungal and Gram-positive bacterial infections. Spätzle, a cytokine-like protein, plays a crucial role in this process. Initially, Spätzle is synthesized as an inactive precursor; upon pathogen recognition by proteins such as GNBP-3 or PGRP-S, a protease cascade is activated, leading to the cleavage and activation of Spätzle. The activated Spätzle then binds to the Toll receptor, initiating a signaling cascade that involves adaptor proteins like Tube and Pelle. This cascade leads to the phosphorylation and subsequent degradation of Cactus, a negative regulator that sequesters in the cytoplasm the transcription factor relish 1 (Rel1), a nuclear factor kappa-light-chain-enhancer of activated B cells (NF-κB)-like transcription factor. Upon activation, this pathway drives the production of AMPs and other immune effectors. In contrast, the Imd pathway is activated by Gram-negative bacterial infections. Bacterial peptidoglycan binds to the transmembrane receptor peptidoglycan recognition protein LC (PGRP-LC), initiating the recruitment of downstream components, including Imd, fas-associated death domain protein (FADD), and death-related ced-3/nedd2-like proteinase (DREDD). These components facilitate the cleavage of the Rel2 transcription factor by DREDD. Cleaved Rel2 translocates to the nucleus, where it induces the expression of effector genes. The Jak-STAT pathway is primarily involved in antiviral immunity and stress responses. Cytokines such as unpaired proteins (Upds) activate a single receptor domeless (Dome), which, in turn, triggers the Jak protein Hopscotch (Hop) and a single STAT transcription factor. This activation induces the transcription of antiviral genes and regulates hemocyte activity. Lastly, the Jnk pathway contributes to stress response and tissue repair, triggered by diverse stress signals, including infections. Extrinsic signals such as the ligand eiger (Egr)—a homolog of tumor necrosis factor alpha (TNFα)—bind to the TNFα receptors grindelwald (Grnd) and wengen (Wgn), activating a kinase cascade. This event involves misshapen (Msn), a mitogen-activated protein 4 kinase (MAP4K); TGF-β-activated kinase 1 (TAK1), an MAP3K; hemipterous (Hep), an MAP2K; and basket (Bsk), the *Drosophila* homolog of Jnk. Ultimately, this signaling cascade phosphorylates the activator protein 1 (AP-1) transcription factor, initiating the specific immune response regulating cell survival, apoptosis, and immunity [37,39]. However, it is important to note that not all instances of immune activation can be fully explained by the pattern recognition concept. For example, certain particles are capable of triggering immune activation even in the absence of microbial patterns on their surface (as discussed below). Preliminary data on this subject were obtained from our studies on Jack bean urease (JBU), the primary urease isoform of the leguminous plant *Canavalia ensiformis*. At first glance, JBU appears to lack recognizable PAMPs. However, treatment of vitellogenic *R. prolixus* females with a sublethal dose of JBU elicited an immune response, impairing the viability of circulating hemocytes and significantly reducing the number of eggs laid [43].

It is worth considering that insect immunity, encompassing both cellular and humoral responses, has evolved under strong selective pressure to effectively counteract diverse pathogens. A key feature of this system is the differential activation dynamics of these two branches: while cellular responses, such as phagocytosis and encapsulation, are triggered rapidly upon pathogen recognition, humoral components, including antimicrobial peptides, typically act later. The speed and specificity of immune activation depend on multiple factors, including hemocyte recruitment dynamics, PAMPs of invading microbes, and the infection route. For instance, oral and systemic infections can elicit distinct immune responses, underscoring the finely tuned adaptability of insect immunity and its evolutionary strategies for pathogen defense [40,44,45].

**Figure 1:**
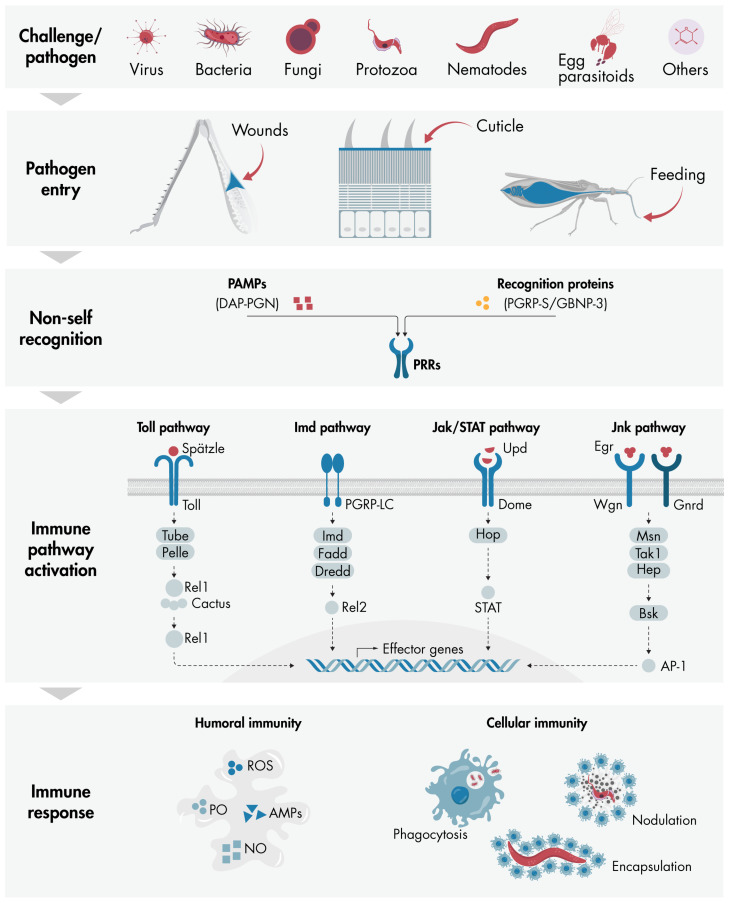
Overview of the key components involved in the initiation and propagation of insect immune responses: The diagram illustrates how various challenges and pathogens—including fungi, viruses, bacteria, protozoa, nematodes, parasitoids, and foreign particles such as Sephadex beads—can penetrate the insect body cavity through wounds, cuticles, or feeding. Once inside, immune responses are triggered via the recognition of PAMPs, such as DAP-PGN, or recognition proteins, such as PGRP-S and GNBP-3, by PRRs present on insect cells. Depending on the type of pathogen, specific immune pathways such as Toll, Imd, Jak/STAT, or Jnk are activated, leading to the nuclear expression of effector genes that coordinate a comprehensive immune response. The key components of each pathway are depicted in the diagram (further details are provided in the main text). The immune response comprises two main components: humoral immunity, involving molecules like AMPs, PO, ROS, and NO; and cellular immunity, which encompasses processes including phagocytosis, nodulation, and encapsulation. AMPs, antimicrobial peptides; DAP-PGN, diaminopimelic acid peptidoglycan; GNBP-3, Gram-negative binding protein 3; NO, nitric oxide; PAMPs, pathogen-associated molecular patterns; PGRP-S, peptidoglycan recognition protein short; PO, phenoloxidase; PRRs, pattern recognition receptors; ROS, reactive oxygen species.

All of these concepts related to immune response in insects are summarized in Figure 1.

## 5. Immune Status Affects Oogenesis, and Vice Versa

Considering that both immunity and reproduction are highly energy-demanding processes, trade-offs are expected, particularly under conditions of nutritional scarcity. With few exceptions, most evidence to date strongly indicates that enhanced immune activity, whether basal or induced, reduces reproductive output, particularly in terms of oogenesis and egg viability. For instance, transcriptomic studies of ovaries and fat bodies from the orthopteran *Locusta migratoria* infected with the bacterium *Micrococcus luteus* during the reproductive preparation period revealed a significant upregulation of *prophenoloxidase 1* (PPO1) and *defensin 3*, an antibacterial peptide, while vitellogenin expression was notably reduced. These findings suggest that, when faced with the trade-off between immune response and reproduction, locusts prioritize resource allocation toward infection resistance over reproductive processes [46]. Conversely, processes such as mating, vitellogenesis, and oogenesis tend to suppress immune responses [4,14].

From a conceptual standpoint, two main approaches can be used to investigate the trade-offs between reproduction and immunity: (a) intervening in the reproductive status of the insect (e.g., mating) and evaluating its impact on the immune response (e.g., number of circulating hemocytes, AMP production, PO activity), and (b) manipulating the insect’s immune status (e.g., challenging with pathogenic or non-pathogenic microorganisms, selecting resistant phenotypes) and then measuring its reproductive output (e.g., number of eggs laid and hatched). This distinction was comprehensively addressed in the excellent review by Schwenke et al. [4]. For the purpose of this review, we primarily focus on the latter approach (Table 1).

Exposure to noninfectious elicitors, such as components of bacterial cell walls, inert molecules like Sephadex beads, heat-killed bacteria, or non-pathogenic organisms, tends to produce consistent reproductive outcomes across the insect orders studied. These challenges typically affect fecundity, leading to a reduced number of eggs being produced (Table 1). For example, in *R. prolixus*, the injection of conidia from the non-pathogenic fungus *Aspergillus niger* resulted in fewer eggs being laid and an increase in atretic ovarian follicles [47]. Similarly, *D. melanogaster* females exposed to lipopolysaccharide (LPS), heat-killed bacteria, or *Escherichia coli* showed a marked reduction in fecundity and egg production [48,49,50,51,52]. When *Anopheles gambiae* females were challenged with LPS or Sephadex beads, they exhibited a reduction in egg production, preceded by a decrease in ovarian protein content and an increase in the number of apoptotic follicles [53,54]. In coleopterans like *Euoniticellus intermedius* and orthopterans such as *Hemideina crassidens*, *Acheta domesticus*, and *Gryllus texensis*, these treatments also resulted in fewer eggs being produced [55,56,57,58], albeit with some variations. In the case of *H. crassidens*, repeated challenges with LPS not only caused a significant reduction in oviposition but also led to a decrease in the lipid and protein contents of the eggs [58]. In *G. texensis*, wounding and exposure to heat-killed bacteria slowed down oviposition and reduced the protein content of the produced eggs [57], while in *A. domesticus*, nylon implantation resulted in smaller eggs [56]. Similarly, when *L. migratoria* females were challenged with the Gram-positive bacterium *M. luteus*, ovarian follicle development was delayed, along with a reduction in vitellogenin expression [46]. In the burying beetle *Nicrophorus vespilloides*, wounding during the breeding period resulted in a reduced number of larvae being produced [59].

For pathogenic organisms, the impact on reproductive output tends to be more pronounced, as a heightened immune response demands greater resource allocation. These resources are redirected from oogenesis and reproduction to drive an immune response. This phenomenon has been observed in several species of Diptera. For instance, challenges with various pathogens on *D. melanogaster* and *Anopheles stephensi* result in reduced fecundity and egg production [49,51,60,61,62,63], while *Drosophila nigrospiracula* and *Aedes aegypti* exhibit a decrease in egg numbers [26,64]. Furthermore, in *A. gambiae*, infections with the protozoan *Plasmodium yoelii nigeriensis* result in an increased number of apoptotic follicles [54]. On the other hand, when the mosquito *Armigeres subalbatus* is infected with the filarial nematode *Brugia malayi*, a decrease in ovarian protein content and extended time to oviposition are observed, without a reduction in the number of eggs produced [65]. In the orthopteran *Teleogryllus oceanicus*, infection with *Serratia marcescens* reduced sperm viability within storage organs [66], while in honeybee queens, a negative correlation was found between the load of sacbrood and black queen cell viruses and the number and viability of sperm stored in the spermatheca [67]. In the hemipteran *Pyrrhocoris apterus*, infections caused by the nematode *Steinernema carpocapsae* or the fungus *Isaria fumosorosea* led to reduced vitellogenin expression [68]. In *R. prolixus*, the immune responses necessary to control the development of the parasites *Trypanosoma cruzi* and *Trypanosoma rangeli* impose significant energetic costs. These demands can compromise the reproductive performance of infected insects by competing for limited energy resources [69]. In *Heliothis virescens*, the injection of freeze-dried cells of the entomopathogenic bacterium *Serratia entomophila* significantly reduced female signaling behavior, indicative of a decrease in the production of pheromones used to attract males. Consequently, this reduction led to diminished mating success [70]. In *T. molitor*, an injection of inactivated *Bacillus cereus* significantly reduced the number of eggs laid, but only in young females. In older females, the injection had no effect on egg production [71]. Nevertheless, some infections with pathogenic organisms do not significantly affect other aspects of reproduction. For instance, in *D. melanogaster*, pre-copulatory reproductive behaviors—such as male courtship, female sexual receptivity, and even mating success—remain unchanged after bacterial infections with *S. marcescens*, *Staphylococcus aureus*, and *Listeria monocytogenes*. Additionally, these reproductive behaviors are unaffected by the genetic activation of the Toll and Imd immune pathways. A similar lack of response was observed when flies were infected with non-pathogenic bacteria, highlighting the complexity of the relationship between infection and reproduction in this species [72].

On the other hand, the immunocompetence of sexually mature adult insects can vary significantly between sexes. In *G. texensis*, reproductively active males prioritize reproduction over immune function, while females may enhance their immune responses during reproductive periods [73]. This sexual dimorphism in immune competence may be linked to the higher risk of sexually transmitted diseases (STDs) in females, particularly due to internal fertilization. In many species, including insects, STDs are more likely to be transmitted from males to females than vice versa, and such infections can severely impact female reproductive fitness [74]. Therefore, the increased immunocompetence observed in reproductively active females may represent an adaptive strategy to mitigate the risks associated with mating, ultimately safeguarding their reproductive success.

Finally, an insect’s immune status can be basally elevated, conferring resistance to a specific pathogen or group of pathogens. For instance, *D. melanogaster* resistant to bacteria and *A. aegypti* resistant to protozoans exhibited reduced fecundity and lower egg viability or hatchability compared to susceptible females [51,75,76,77]. Likewise, the lepidopteran *Plodia interpunctella* resistant to a granulovirus showed decreased egg viability [78]. In contrast, a different pattern was observed in *T. molitor*. In this species, an elevated immune status, phenotypically evident as enhanced pigmentation associated with higher PO activity, did not negatively affect fecundity [79].

**Table 1:** Summary of the effects of immune status on the reproductive output.

Immune Status	Order	Species	Challenge/ Phenotype	Effect on Reproduction
Challenged with non-pathogenic organisms/ noninfectious elicitors	Hemiptera	*Rhodnius* *prolixus*	*Aspergillus niger* (fungus)	Reduced number of eggs, increased number of atretic follicles [47].
	Orthoptera	*Acheta* *domesticus*	Nylon filament	Reduced number and size of eggs [56].
		*Gryllus* *texensis*	Wound, heat-killed (HK) *Serratia marcescens* (bacterium)	Reduced oviposition rate and egg protein content [57].
		*Hemideina* *crassidens*	Lipopolysaccharide (LPS)	Reduced number of eggs and egg protein content [58].
		*Locusta* *migratoria*	*Micrococcus luteus* (bacterium)	Reduced ovarian development, reduced vitellogenin expression [46].
	Diptera	*Drosophila* *melanogaster*	LPS, HK bacteria, *Escherichia coli*, *M. luteus* and *Pectinobacterium carotovorum carotovorum* (bacteria)	Reduced fecundity and number of eggs. No effect on pre-copulatory behaviors [48,49,50,51,52,72].
		*Anopheles* *gambiae*	LPS, Sephadex beads	Reduced ovarian protein content, reduced number of eggs, increased number of apoptotic follicles [53,54].
	Coleoptera	*Euoniticellus* *intermedius*	LPS	Reduced number of eggs [55].
		*Nicrophorus* *Vespilloides*	Wound	Reduced number of larvae [59].
Challenged with pathogens	Hemiptera	*Pyrrhocoris* *apterus*	*Steinernema carpocapsae* (nematode), *Isaria fumosorosea* (fungus)	Reduced vitellogenin expression [80].
	Orthoptera	*Teleogryllus* *oceanicus*	*S. marcescens*	Reduced viability of stored sperm [66].
	Diptera	*Drosophila* *melanogaster*	*Providencia rettgeri*, *S. marcescens*, *Staphylococcus aureus*, and *Listeria monocytogenes* (bacteria); *Beauveria bassiana* (fungus); *Asobara* *tabida* (parasitoid wasp)	Reduced fecundity. No effect on pre-copulatory behaviors [49,51,60,61,62,72].
		*Drosophila* *nigrospiracula*	*Macrocheles subbadius* (acarus)	Reduced number of eggs [64].
		*Aedes* *aegypti*	*Enterobacter cloacae* (bacterium)	Reduced vitellogenin expression and number of eggs [26].
		*Anopheles* *gambiae*	*Plasmodium yoelii* *nigeriensis* (protozoon)	Increased number of apoptotic follicles [54].
		*Anopheles* *stephensi*	*Plasmodium yoelii* *nigeriensis*	Reduced fecundity [63].
		*Armigeres* *subalbatus*	*Brugia malayi* (nematode)	Reduced ovarian protein content and increased time to oviposition [65].
	Coleoptera	*Tenebrio* *molitor*	Inactivated *Bacillus cereus*	Reduced number of eggs [71].
	Lepidoptera	*Heliothis* *virescens*	Death cells of *Serratia entomophila*	Reduced signal behavior by females (production of pheromones for male attraction) [70].
	Hymenoptera	*Apis* *mellifera*	Sacbrood and black queen cell virus	Lower sperm viability and fewer sperm in the spermatheca [67].
Increased resistance to pathogens	Diptera	*Drosophila* *melanogaster*	Resistance to *Pseudomonas aeruginosa* and *Providencia rettgeri* (bacteria)	Reduced fecundity and egg viability [51,75,76].
		*Aedes* *aegypti*	Resistance to *Plasmodium gallinaceum*	Reduced fecundity and hatchability [77].
	Lepidoptera	*Plodia* *interpunctella*	Resistance to *Plodia interpunctella* granulovirus	Reduced egg viability [78].
	Coleoptera	*Tenebrio* *molitor*	Increased pigmentation (equivalent to phenoloxidase activity)	No effect on fecundity [79].

## 6. Neuroendocrine and Endocrine Regulators

In vertebrates, a dynamic interplay exists between the immune system, neuroendocrine/endocrine system, and reproduction. The immune system, through cytokines produced by primary lymphoid organs, provides feedback to neuroendocrine centers such as the hypothalamus and pituitary gland, modulating hormone synthesis or release. Hormones, in turn, exert their effects either directly, by binding to specific hormone receptors, or indirectly, by altering cytokine and growth factor levels, which target immune cells and regulate their secretory activity. This bidirectional interaction not only governs immune and endocrine regulation but also influences broader physiological processes, including development and reproduction [81,82]. For instance, sex steroid hormones, such as estrogens, androgens, and progesterone, impact both innate and adaptive immunity, maintaining a delicate balance between organismal defense and reproduction [83,84]. While the immune–endocrine crosstalk in vertebrates has been extensively studied, recent research indicates that similar interactions also occur in insects, albeit through mechanisms uniquely adapted to their physiology. Insects rely mainly on their neuroendocrine and endocrine systems to regulate both reproduction and immune responses to pathogens, effectively optimizing the allocation of resources between self-maintenance and species propagation [36,85,86]. Among insect hormones, JH and ecdysone stand out as two of the most critical regulators.

Due to its lipophilic nature, JH readily crosses the cell membrane to bind to the nuclear receptor Methoprene-tolerant (Met), a member of the conserved basic Helix-Loop-Helix-Per-ARNT-Sim (bHLH-PAS) transcription factor family. Seven epoxidated JH homologs, collectively referred to here as JH, and a non-epoxidated methyl farnesoate (MF) have been identified in various arthropods, all functioning through interaction with Met [87]. The JH–Met interaction promotes the formation of a heterodimer between Met and Taiman (Tai), another bHLH-PAS protein, which subsequently activates downstream signaling pathways [6]. Additionally, JH has been proposed to interact with a hypothetical membrane receptor, although its identity remains undefined [88]. However, experimental evidence suggests that membrane-associated JH signaling can independently activate the G protein-coupled receptor–receptor tyrosine kinase (GPCR-RTK) pathway [6].

Ecdysone, a steroid hormone analogous to mammalian estrogens and androgens, is converted in peripheral tissues to 20E, the most biologically active form of ecdysteroid. The canonical ecdysone signaling pathway involves the binding of 20E to the ecdysone receptor (EcR)–ultraspiracle (USP) protein complex, which, in turn, mediates the downstream effects of 20E [89].

Juvenile hormone and 20E coordinate development and reproduction while also influencing immune processes, with the impact of this coordination varying across species. In cellular immunity, early studies in *D. melanogaster* demonstrated that the neuroendocrine system modulates the transformation of hemocytes into lamellocytes [90,91,92]. In the hemipteran *Halys dentata*, the lepidopteran *Galleria mellonella*, and the orthopteran *L. migratoria*, the neuroendocrine system plays a pivotal role in hemocyte proliferation and release [93,94,95]. In *R. prolixus*, a well-established model insect for studies on neuroendocrine/endocrine-mediated reproduction [96], hormones regulate both the amount of hemolymph available and the cyclical changes in the types and numbers of hemocytes [97]. For example, specifically regarding developmental hormones, early studies on the blattodean *Blattella germanica* demonstrated that injecting a JH analogue decreases the plasmatocyte population without affecting granulocytes [98]. In *D. melanogaster*, ecdysteroid signaling promotes hemocyte functions such as actin dynamics, motility, phagocytosis, and chemotaxis toward damaged epithelia [99,100], while in the dipteran *Neobelliera bullata*, JH suppresses the 20E-induced nodulation response [101]. Moreover, ecdysteroids promote increased phagocytic activity in hemocytes and upregulate the expression of *leucine-rich repeat protein 9* (LRIM9), a protein that activates the complement system in the fat body of *A. gambiae* [102]. The complement system is part of the innate immune response and helps identify and eliminate pathogens, such as bacteria and parasites. All of these studies exemplify the long-established link between reproductive hormones and immune effectors in insects, highlighting their roles not only in hemocyte proliferation and differentiation but also in the regulation of immune processes.

In terms of humoral immunity, 20E enhances immune responses by enabling *D. melanogaster* cells to activate AMP genes, such as *Diptericin* and *Drosomycin* [103,104,105]. Similarly, 20E induces the expression of hemolin, a hemolymph protein belonging to the immunoglobulin superfamily, in the fat body of diapausing pupae of the lepidopteran *Hyalophora cecropia* [106], as well as reducing antibacterial activity in the diapausing larvae of the blowfly *Calliphora vicina* [107]. While 20E can suppress certain immune functions, such as downregulating Toll pathway components and AMP production during *D. melanogaster* metamorphosis [108], JH generally acts as a more consistent immunosuppressant. For example, in the lepidopteran *Manduca sexta*, JH inhibits PO production, reducing cuticular melanization [109]. Similar effects are observed in the coleopteran *T. molitor*, where JH decreases PO levels and suppresses encapsulation responses [110,111]. Interestingly, in the lepidopteran *Spodoptera exigua* and the coleopteran *Tribolium castaneum*, JH modulates cellular immunity by suppressing hemocyte behaviors through mechanisms independent of its nuclear receptor Met, likely acting via a hypothetical membrane receptor [112,113].

Taken together, the evidence suggests that 20E generally acts as a positive regulator of innate immunity, whereas JH functions as an immunosuppressant during successful reproduction. Their antagonistic interplay contributes to balancing various physiological responses [114]. Beyond immunity, this dynamic is also observed in other physiological processes, particularly during development and reproduction [115]. In the context of reproduction, the interaction between these hormones exhibits considerable variability. Juvenile hormone is critical for vitellogenesis in many insect orders, including Orthoptera, Blattodea, Coleoptera, and Hemiptera [32]. This species-specific variability in JH responses enables insects to adapt to diverse environmental conditions and life history strategies. Similarly, ecdysteroids are key regulators of vitellogenesis, but their effects differ across species. In most lepidopteran and dipteran species, ecdysteroids stimulate vitellogenesis, whereas in other orders they act as a signal for its termination. These findings highlight the complex and diverse mechanisms underlying hormonal regulation in insect reproduction [6]. This intricate hormonal interplay not only ensures the precise regulation of immunity and reproduction but also underscores its critical role in balancing energy allocation, enabling insects to adapt and thrive in diverse environmental and physiological contexts.

Circulating JH and/or ecdysteroid titers are high in mated females of several insect species [23,116,117]. Mating influences various aspects of immune function, including mechanisms of cellular defenses and PO activity [8,110,118,119,120,121]. However, while mating generally downregulates immune responses in females across most insect species, some show no differences in their immune defenses between mated and virgin females [7]. In the female ground cricket *Allonemobius socius*, frequent copulation leads to a decrease in circulating hemocyte numbers and reduced bacteriolytic activity in the hemolymph [120]. Similarly, in the mealworm beetle *T. molitor*, mating induces the *corpora allata* to release JH, which plays a role in downregulating PO activity [110]. On the other hand, mating can challenge the female immune system by introducing foreign substances and potential infections. As a result, immune activation typically reduces reproductive investment, leading to effects such as diminished ovarian protein reserves, apoptosis of follicle cells, and reduced egg production, prioritizing immune defense and recovery over reproduction [54,110,118,120,122]. A transcriptomic analysis using microarrays revealed that females of *D. melanogaster* activate numerous genes necessary for producing fertile eggs after a single mating. However, a second mating has a much smaller impact on these genes. Instead, in remated females, many immune-related genes show differential expression, suggesting that immune system activation may account for much of the cost of mating [123]. More recently, it has been shown that mating and the receipt of male seminal fluid proteins result in reduced resistance to systemic bacterial infection, which can be attributed to JH signaling, promoting oocyte development and yolk protein synthesis and deposition [124]. In *Spodoptera litura*, post-mating behavioral and physiological changes in females seem to be linked to modifications in gene expression, which primarily enhance reproduction-related gene activity while suppressing immunity-related gene activity, at least shortly after mating [125].

Ceratotoxin, a female-specific antibacterial peptide in *Ceratitis capitata*, is produced in the accessory glands and secreted into the reproductive tract, where it provides protection against microbial infections, particularly Gram-negative bacteria [126]. Unlike many immune peptides that are upregulated in response to infection, ceratotoxin production is primarily triggered by mating rather than pathogen presence. Juvenile hormone regulates its synthesis and secretion, ensuring its production when the female is sexually mature and actively laying eggs. By linking ceratotoxin production to JH levels and mating status, *C. capitata* enhances reproductive success by safeguarding the reproductive tract precisely when it is most vulnerable, such as after mating and during oviposition [127].

As is widely known, blood feeding is an integral behavior of mosquitoes to acquire the nutritional resources needed for reproduction, a process driven by 20E. In turn, the activation of 20E signaling has the capacity to prime an immune response during this process, which can limit bacterial and malarial parasite survival in *A. gambiae* [102]. However, upon blood feeding alone, hemocytes exhibit elevated levels of key components involved in the complement system and the melanization process. In addition, hemocytes show changes in recognized markers of immune cell activation, increasing in number, size, granularity, Ras-mitogen-activated protein kinase (Ras-MAPK) signaling, and altered cell surface moieties. These findings demonstrate that the innate immune response in mosquitoes is naturally activated by the physiological process of blood feeding [128]. Moreover, during the vitellogenic phase of *A. aegypti*, the ortholog of *D. melanogaster* Pirk, designated as Pirk-like, is upregulated by the 20E-EcR signaling pathway in the fat body, after which it inhibits the initiation of Imd signaling, preventing the nuclear translocation of the transcriptional factor Rel2, thereby inhibiting amyloid formation. This is an essential action for maintaining normal yolk protein production and fertility [26].

An active immune response requires significant energy, necessitating the reallocation of nutrients to enhance resistance against infections. Insulin signaling plays a pivotal role as a global regulator of metabolism, responding to nutrient availability and energetic demands to support immune functions. In mosquitoes, a key regulator of egg development following blood feeding is the release of ILPs from the brain. These ILPs also stimulate hemocyte proliferation, which constitutes the first line of defense against infection. After a blood meal, the number of circulating hemocytes increases, potentially enhancing the female’s ability to combat invading pathogens. Interestingly, genes encoding immune effectors, such as AMPs, are not transcriptionally activated. It is hypothesized that this lack of immune gene upregulation may represent an adaptation to minimize the risk of inflammatory responses that could lead to tissue damage in mosquitoes [9,10]. In addition, using a genetic screening approach, an insulin response was shown to be a key component of the immune response to arboviral infections in some insects. Insulin reduces West Nile virus (WNV) replication in *D. melanogaster* and decreases the titers of WNV, Zika, and dengue viruses in mosquito cells. Mechanistically, insulin signaling activates the Jak/STAT pathway through MAPK/ERK activation, an essential pathway mediating insulin’s effects on metabolism and cell function. Furthermore, insulin priming of adult female *Culex* mosquitoes through a blood meal significantly reduced WNV infection, highlighting the pivotal role of insulin signaling in the antiviral defenses of insects against human pathogens [129].

In vitro experiments also demonstrated that insulin acts synergistically with a hemolymph factor to specifically promote the mitotic division of granulocytes in the lepidopteran *Bombyx mori* [130]. Additionally, in this species, the release of plasmatocytes from hematopoietic organs is regulated by insulin-mediated signaling and its downstream pathways, including PI3K/Akt and MAPK/Erk signals [131].

Octopamine, a biogenic amine involved in locomotion, metabolism, reproduction, and stress responses [132], is released upon immune induction, positioning it as a strong candidate for regulating lipid and carbohydrate utilization post-infection [133]. Also, octopamine and 5-hydroxytryptamine (serotonin) seem to stimulate nodule formation in *Periplaneta americana* and *G. mellonella*. However, these particular amines had no effect when co-injected with LPS into locusts [133,134]. Interestingly, although adipokinetic hormone (AKH), the primary regulator of energy mobilization, has been implicated in insects’ immunity [135,136], its release during an immunological event remains to be demonstrated [137]. It has been reported that AKH appears to exert its effects, at least partially, through its impact on lipid metabolism. A lipid-associated form of apolipophorin-III (apoLp-III) is induced by AKH, and this may be involved in the activation of PPO by LPS [134,138]. Moreover, tachykinin peptides have been identified as contributors to insects’ immunity. In *T. molitor*, tachykinins contribute to both cellular and humoral immune responses, with their effects being time- and dose-dependent [139].

## 7. Dual Role of the Yolk Protein Precursors Vitellogenin and Lipophorin

Vitellogenin was initially described as a yolk protein precursor dedicated solely to egg development. However, subsequent studies have demonstrated its expression in males and sexually immature animals, indicating that vitellogenin’s functions extend beyond serving as an energy reserve for nourishing developing embryos [33,140,141]. Similarly, lipophorin, another yolk protein precursor primarily recognized for its role in lipid transport, has been shown to possess immune-related functions. Specifically, it acts as a pattern recognition molecule, binding and neutralizing microbial cell wall components such as lipopolysaccharides, lipoteichoic acids, and beta-1,3-glucans [142]. In addition, lipophorin stimulates the expression and activity of AMPs, regulates the PO cascade, contributes to hemolymph clotting, and modulates hemocyte adhesion and phagocytosis [143]. Together with vitellogenin, lipophorin plays a crucial role in the mechanisms of transgenerational immune priming in insects. These yolk protein precursors can bind bacteria or their fragments, facilitating their transmission both horizontally and vertically [13]. For instance, in honeybees, vitellogenin binds to various bacteria, including *Paenibacillus* larvae and *E. coli*, with binding specificity assessed using bovine serum albumin as a negative control. Moreover, in the ovaries, vitellogenin interacts with PAMPs and facilitates the transport of fluorescently labeled *E. coli* cell wall fragments into developing eggs [144]. This mode of transmission elicits specific immune responses not only within the same generation but also across subsequent ones, underscoring their role in inherited immunity. In addition, in the same species, vitellogenin functions as an antioxidant, enhancing oxidative stress tolerance in live cells and increasing lifespan. Notably, it exhibits cell- and membrane-binding activity, with a preference for damaged or dead cells. While vitellogenin binds directly to phosphatidylcholine liposomes, it shows a higher affinity for liposomes containing phosphatidylserine, a lipid typically confined to the inner leaflet of cell membranes but exposed in damaged cells [145,146]. Furthermore, in the hymenopteran *Apis mellifera*, the reduction in hemocyte count in foragers is linked to decreased hemolymph zinc levels due to suppressed vitellogenin synthesis regulated by JH. In this context, vitellogenin serves as the primary zinc carrier in the hemolymph, playing a crucial role in maintaining normal hemocyte function [147].

In *A. gambiae*, a major vector for malaria, both vitellogenin and lipophorin fulfill dual roles in reproduction and immunity. After a female mosquito takes a blood meal from a malaria-infected host, it acquires the necessary nutrients for egg development while also potentially becoming infected with *Plasmodium* spp. parasites. As the parasites transit through the mosquito’s midgut, they face a robust innate immune response, primarily regulated by hemocytes. In this scenario, lipophorin and vitellogenin perform distinct yet complementary functions. Lipophorin is crucial for oogenesis and supports the normal expression of vitellogenin after an infectious blood meal. Meanwhile, vitellogenin not only contributes to oogenesis but also modulates the immune response by negatively regulating the binding of the antiparasitic factor thioester-containing protein 1 (TEP1) to the parasites. Consequently, the depletion of lipophorin indirectly affects parasite survival through its impact on vitellogenin levels. Additionally, the NF-κB factors Rel1 and Rel2 have shown to suppress vitellogenin expression in response to an infectious blood meal [148]. In *Laodelphax striatellus*, the vector of rice stripe virus (RSV), vitellogenin plays a pivotal role in viral transmission. RSV, which relies entirely on this insect for plant-to-plant transmission, can also be vertically transmitted from mother insects to their offspring. A critical step in both horizontal and vertical transmission involves the passage of RSV through the hemolymph. In *L. striatellus*, vitellogenin binds to RSV, protecting the virus and facilitating its transport. Notably, studies have identified unique, tissue-specific forms of vitellogenin that are crucial for virus transmission, providing novel insights into the role of vitellogenin in virus–vector interactions, and highlighting the often-overlooked vector phase of plant virus transmission [149]. Interestingly, in *P. apterus* females, infection by the nematode *S. carpocapsae* significantly upregulates vitellogenin mRNA and protein expression in males, while in females, both mRNA and protein levels are reduced. These findings suggest that vitellogenin synthesis is important for immune defense but insufficient for reproduction in infected females, likely conserving energy to manage the infection. Additionally, purified vitellogenin inhibits the growth of *Xenorhabdus* spp., whereas its effect on the fungus *I. fumosorosea* is less pronounced, emphasizing the critical role of vitellogenin in immune defense and its potential specificity of activity [80].

In the greater wax moth *G. mellonella*, oenocytoids release nucleic acids upon immune activation. Apolipophorin-III, a multifunctional protein, not only facilitates lipid transport but also mediates nucleic acid-driven hemocyte activation, enhancing innate immune responses, inducing coagulation, and prolonging survival during infection [150]. Interestingly, *D. melanogaster* lacks the *apoLp-III* gene, underscoring significant differences in immune mechanisms among insect species and highlighting the critical need for comparative studies across diverse insect taxa. However, although apoLp-III is the most extensively studied apolipoprotein in insect immunity, evidence suggests that apoLp-III works together with apolipophorin-I (apoLp-I) and apolipophorin-II (apoLp-II) in coordination to mount a unified defense against pathogens [143].

## 8. Conclusions and Subjects for Future Research

**Figure 2:**
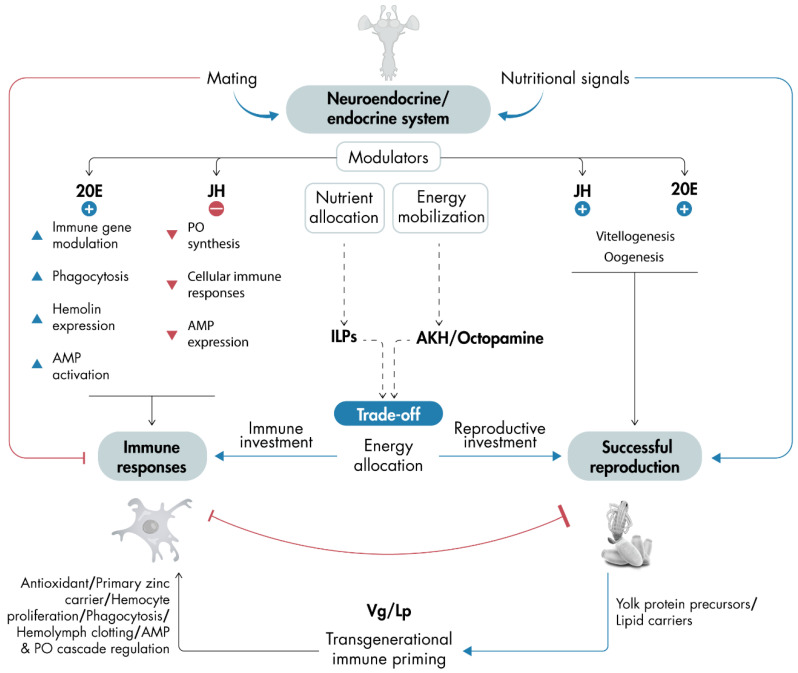
Schematic representation of a model illustrating how mating and nutritional signals influence immune responses and reproduction, either directly or through the regulation of the neuroendocrine and endocrine systems: The neuroendocrine and endocrine systems, governed by neuropeptides and hormones, balance these processes. In general, JH and 20E promote reproduction, while 20E stimulates immune responses and JH inhibits them (the figure highlights key events regulated by JH and 20E signaling). The neuroendocrine and endocrine systems also govern nutrient allocation and energy mobilization via neuropeptides like ILPs and AKH/octopamine. These signals mediate trade-offs, determining whether resources are allocated to immune responses or successful reproduction. Typically, active immune responses suppress reproduction, while active reproduction may inhibit appropriate immune defense. If reproduction is activated and successful, it triggers a massive synthesis of yolk protein precursors, such as Vg and Lp, which also contribute to immune responses (the figure displays key events regulated by Vg and Lp). One of the key roles linking immunity and reproduction is the involvement of Vg and Lp in transgenerational immune priming. This model emphasizes the balance among neuroendocrine and endocrine regulation, immune responses, and reproduction. Dashed arrows indicate speculative processes, while solid arrows represent pathways or interactions supported by scientific evidence. 20E, 20-hydroxyecdysone; AKH, adipokinetic hormone; ILPs, insulin-like peptides; JH, juvenile hormone; Lp, lipophorin; OA, octopamine; Vg, vitellogenin.

The relationship between immunity and reproduction in insects is profoundly interconnected, with both processes sharing regulatory mechanisms. These mechanisms, primarily related to energy demands and hormone regulation, enable insects to adapt efficiently to diverse challenges. This interdependence is essential for survival and species propagation, shaping evolutionary adaptations and responses to ecological pressures (Figure 2). Recent advancements in omics technologies have significantly enhanced our understanding of this complex relationship, uncovering species-specific variations and offering deeper insights into how insects allocate resources according to their physiological states.

The critical role of the neuroendocrine and endocrine systems in regulating specific processes based on the insect’s needs has been a central focus of this review. The suppression of immune responses following mating, alongside the reallocation of nutrients toward reproduction or immune function, highlights the remarkable flexibility of the insect neuroendocrine and endocrine systems. This adaptability, primary modulated by JH, 20E, and ILPs, enables insects to balance competing physiological demands (Figure 2). Given the species-specific variation in neuroendocrine/endocrine regulation, further investigation into this aspect remains a key challenge for the field.

A particularly intriguing aspect of the immunity–reproduction interplay is the multifunctionality of the recognized yolk protein precursors, vitellogenin and lipophorin. These macromolecules are not only relevant to reproduction but also play significant roles in immunity and pathogen transmission, underscoring the complex and dynamic connections between immune responses and reproductive processes in insects.

Current research efforts aim to identify the “biological actors” that influence energy allocation decisions, which are crucial in balancing immune defense and reproduction in response to stressors. A significant knowledge gap persists regarding the neuroendocrine/endocrine regulation of energy mobilization during such challenges. Notably, octopamine is released during immune responses and plays a pivotal role in regulating energy mobilization under these conditions. Further research is needed to clarify how octopamine governs lipid and carbohydrate utilization following infection in insects. In this new era, accessing protocols and addressing methodological challenges have become more streamlined, thanks to open-access resources and technological advancements. This increased accessibility enables researchers to integrate diverse methodologies and design more robust experiments, particularly those involving transcriptomic and proteomic analyses to study hormonal interactions, RNA interference (RNAi) to investigate physiological implications, and comparative studies across insect species to explore evolutionary perspectives. Advancing our understanding of how these biological actors mediate resource allocation will deepen insights into the physiological mechanisms underlying insect survival and species propagation. For example, the mobilization of lipids and carbohydrates during immune challenges could limit the energy available for reproduction or immune function, effectively reducing pest populations. Additionally, the dual roles of vitellogenin and lipophorin in both reproduction and immunity offer unique opportunities for intervention. For instance, inhibiting the production or function of these lipoproteins could simultaneously impair egg development and immune responses, providing a dual-action strategy for pest control.

Altogether, future research should prioritize cross-species comparisons to uncover universal mechanisms linking immunity and reproduction, alongside investigating the functional roles of vitellogenin and lipophorin in immune response. The integration of omics tools will be essential for uncovering the molecular mechanisms underlying these processes.

## Data Availability

No new data were created or analyzed in this study. Data sharing is not applicable to this article.

## Abbreviations

The following abbreviations are used in this manuscript:

20E	20-Hydroxyecdysone
AKH	Adipokinetic hormone
AMPs	Antimicrobial peptides
AP-1	Activator protein 1
ApoLp	Apolipophorin
bHLH-PAS	Basic Helix-Loop-Helix—Per-ARNT-Sim (bHLH-PAS)
Bsk	Basket
DAP-PGN	Diaminopimelic acid peptidoglycan
Dome	Domeless
DREDD	Death-related ced-3/nedd2-like proteinase
EcR	Ecdysone receptor
Egr	Eiger
FADD	Fas-associated death domain
FAS2	Fatty acid synthase 2
GNBP	Gram-negative binding protein
GPCR	G protein-coupled receptor
Grnd	Grindelwald
Hep	Hemipterous
Hop	Hopscotch
ILPs	Insulin-like peptides
Imd	Immune deficiency
Jak-STAT	Janus kinase–signal transducer and activator of transcription
JBU	Jack bean urease
JH	Juvenile hormone
Jnk	c-Jun N-terminal kinase pathway
Lp	Lipophorin
LPS	Lipopolysaccharide
MAPK	Mitogen-activated protein kinase
Met	Methoprene-tolerant
MF	Methyl farnesoate
Msn	Misshapen
NF-κB	Nuclear factor kappa-light-chain-enhancer of activated B cells
NO	Nitric oxide
OA	Octopamine
PAMPs	Pathogen-associated molecular patterns
PGRP-LC	Peptidoglycan recognition protein LC
PGRP-S	Peptidoglycan recognition protein short
PO	Phenoloxidase
PPO1	Prophenoloxidase 1
PRRs	Pattern recognition receptors
Ras-MAPK	Ras-mitogen-activated protein kinase
Rel1	Relish transcription factor 1
Rel2	Relish transcription factor 2
ROS	Reactive oxygen species
RSV	Rice stripe virus
RTK	Receptor tyrosine kinase
Tai	Taiman
TAK1	TGF-β-Activated kinase 1
TEP1	Thioester-containing protein 1
TNF	Tumor necrosis factor
Upds	Unpaired proteins
USP	Ultraspiracle
Vg	Vitellogenin
Wgn	Wengen
WNV	West Nile virus
